# 30-day in-hospital mortality after acute myocardial infarction in Tuscany (Italy): An observational study using hospital discharge data

**DOI:** 10.1186/1471-2288-12-170

**Published:** 2012-11-08

**Authors:** Chiara Seghieri, Stefano Mimmi, Jacopo Lenzi, Maria Pia Fantini

**Affiliations:** 1 Scuola Superiore Sant’Anna, Laboratorio Management e Sanità, Institute of Management, Piazza Martiri della Libertà 33, Pisa, 56127, Italy; 2 Department of Medicine and Public Health, University of Bologna, Bologna, Italy

**Keywords:** Myocardial infarction, Mortality, Cardiovascular risk, Medical records

## Abstract

**Background:**

Coronary heart disease is the leading cause of mortality in the world. One of the outcome indicators recently used to measure hospital performance is 30-day mortality after acute myocardial infarction (AMI). This indicator has proven to be a valid and reproducible indicator of the appropriateness and effectiveness of the diagnostic and therapeutic process for AMI patients after hospital admission. The aim of this study was to examine the determinants of inter-hospital variability on 30-day in-hospital mortality after AMI in Tuscany. This indicator is a proxy of 30-day mortality that includes only deaths occurred during the index or subsequent hospitalizations.

**Methods:**

The study population was identified from hospital discharge records (HDRs) and included all patients with primary or secondary ICD-9-CM codes of AMI (ICD-9 codes 410.xx) that were discharged between January 1, 2009 and November 30, 2009 from any hospital in Tuscany. The outcome of interest was 30-day all-cause in-hospital mortality, defined as a death occurring for any reason in the hospital within 30 days of the admission date. Because of the hierarchical structure of the data, with patients clustered into hospitals, random-effects (multilevel) logistic regression models were used. The models included patient risk factors and random intercepts for each hospital.

**Results:**

The study included 5,832 patients, 61.90% male, with a mean age of 72.38 years. During the study period, 7.99% of patients died within 30 days of admission. The 30-day in-hospital mortality rate was significantly higher among patients with ST segment elevation myocardial infarction (STEMI) compared with those with non-ST segment elevation myocardial infarction (NSTEMI). The multilevel analysis which included only the hospital variance showed a significant inter-hospital variation in 30-day in-hospital mortality. When patient characteristics were added to the model, the hospital variance decreased. The multilevel analysis was then carried out separately in the two strata of patients with STEMI and NSTEMI. In the STEMI group, after adjusting for patient characteristics, some residual inter-hospital variation was found, and was related to the presence of a cardiac catheterisation laboratory.

**Conclusion:**

We have shown that it is possible to use routinely collected administrative data to predict mortality risk and to highlight inter-hospital differences. The distinction between STEMI and NSTEMI proved to be useful to detect organisational characteristics, which affected only the STEMI subgroup.

## Background

Coronary heart disease, and in particular acute coronary syndromes (ACS), is the leading cause of mortality in the world [1]. Of the syndromes that comprise ACS, ST-segment elevation acute myocardial infarction (STEMI) and non-ST segment elevation acute myocardial infarction (NSTEMI) have been reported to have differing short-term prognosis and response to therapies [2,3].

Although important steps have been taken in the care of patients with these conditions, a substantial proportion of patients do not receive effective treatments, and population-based studies have been called for to evaluate the successful translation of evidence-based medicine into clinical practice [4]. As a result, emphasis has increasingly been placed on the quality of hospital services and on the equity of access to effective care for patients with acute myocardial infarction (AMI).

One of indicators that has proved to be a valid and reproducible indicator of the appropriateness and effectiveness of the diagnostic and therapeutic process that begins with the admission of patients with this condition is 30-day mortality [5-10].

To assess the performance of health care services, the Italian Tuscany region introduced in 2005 an evaluation system based on the systematic collection of indicators to examine the efficiency, effectiveness and appropriateness of care for a number of clinical conditions. Indicators were determined from administrative data sources, that are increasingly being used to examine the quality of care and patient outcomes and to compare hospital performance in Western countries [11,12]. One of the outcome indicators recently included in the Tuscan performance evaluation system is 30-day in-hospital mortality after AMI, that is a proxy of 30-day mortality including only deaths occurred during the index or subsequent hospitalizations.

The aim of this study was to examine the determinants of inter-hospital variability in 30-day in-hospital mortality after AMI in Tuscany, using an analytical strategy to determine the influence of patient and organisational characteristics.

Specifically, because patients’ risk factors may vary across hospitals, the inter-hospital comparison was carried out using risk adjustment methods to control for the patient case mix and by stratifying the patients according to the presence or absence of ST-segment elevation. Multilevel models were used, as has been strongly recommended [6], to take into account the effects of clustering of patients into hospitals and of hospital characteristics, including organisational variables, that may affect short-term mortality.

## Methods

The study population was identified from hospital discharge records (HDRs), and included all patients with primary or secondary ICD-9-CM codes of AMI (ICD-9 codes 410.xx) that were discharged between January 1, 2009 and November 30, 2009 from any hospital in Tuscany.

Data on hospital discharges in Tuscany region are routinely entered at hospital level into the HDR database. The hospital information system collects demographic information and ICD-9-CM diagnoses and procedures. These data are sent every 3 months for quality check to the Regional Health Information System Office that gives feedback to the hospitals on logical inconsistencies and missing data. Data are then corrected, completed and sent back the Regional Health Information System Office. As a results of this process, only a limited percentage of records are discarded, given that hospital reimbursement is based on these routine data.

The study was carried out in compliance with the Italian law on privacy (Art. 20–21, DL 196/2003) and the regulations of the Health Authorities of Tuscany Region on data management. Data were anonymized at the Regional Health Information System Office where each patient was assigned a unique identifier that is the same for all administrative databases. This identifier does not allow to trace the patient’s identity and other sensitive data. When anonymized administrative data are used to inform health care planning activities, the study is exempt from notification to the Ethics Committee provided written consent is obtained to use patient’s information stored in the hospital databases.

Records were excluded from the analysis on the basis of the following criteria:

1. Admissions preceded by a diagnosis of AMI in the preceding 8 weeks. This was done to exclude sequelae of a previous episode, according to the ICD-9-CM classification which defines "8 weeks" as the limit for a single "episode of care";

2. Admission lasting less than 2 days with discharge home;

3. Transfers from other hospitals;

4. Patients not resident in Tuscany;

5. Patients aged under 18 years or more than 100 years;

6. A diagnostic code 410.9 (myocardial infarction of unspecified site).

If patients were transferred, mortality was attributed to the hospital to which the patient was initially admitted [12].

### Data

The outcome of interest was 30-day all-cause in-hospital mortality, defined as a death occurring for any reason within 30 days of the admission date during the index hospitalization or in any subsequent hospitalization. The follow-up was performed through a deterministic record linkage procedure of hospital discharge records from all Tuscan hospitals, using the unique patient identifier. The period of follow-up was determined as 30 days from the index admission. Our outcome differs from 30-day mortality used by other authors that also include deaths occurring out of the hospital, within 30 days of the index admission [13,14]. We could not analyse out-of-hospital deaths because they can be obtained from the mortality registry database available to researchers only after a time lag of several years.

Hospitals were categorised according to a presence or absence of the cardiac catheterisation laboratory, defined as “a laboratory operating in a hospital with in-house cardiovascular surgical support, in which both diagnostic and therapeutic procedures are performed on the heart and great vessels for a wide variety of cardiovascular diseases” [15].

Information on the following variables, that might influence the outcome of interest and whose distribution may differ across hospitals, was retrieved from the HDRs: patient age, sex and comorbidities. The ICD-9-CM codes were used to define the presence or absence of specific comorbidities (hematologic diseases, previous AMI episodes, cerebrovascular diseases, vascular diseases, chronic nephropathies, tumours, diabetes, hypertensive disease, other forms of ischemic heart disease, conduction disorders and cardiac dysrhythmias, and chronic obstructive pulmonary disease) at the index admission and in the previous 2 years.

According to the ICD-9-CM classification that was adopted in Italy in 2007, the type of ST-segment elevation was determined as follows: patients with 410.1x–410.6x or 410.8x were defined as STEMI; patients with 410.7x were defined as NSTEMI. Codes 410.9 were excluded from the analyses [16].

The validity of the ICD-9-CM coding in the Tuscany region compared with clinical data was examined in separate samples collected in the framework of AMI-Florence registry data [17] and in the IN-ACS study [18]. Sensitivity, specificity and PPV were 90.4%, 71.9%, 69.5% in AMI-Florence (2009 data) and 98%, 90.4% and 96% in IN-ACS study, supporting the validity of the codes.

### Statistical analysis

We determined the differences in patient characteristics according to the two hospital types (presence/absence of a cardiac catheterisation laboratory) using Pearson’s *χ*^*2*^ test or the Student’s *t*-test as appropriate. Comparison of 30-day in-hospital mortality rates between patients with STEMI and NSTEMI was performed using Pearson’s *χ*^*2*^ test.

Because of the hierarchical structure of our data, with patients clustered into hospitals, we fitted random-effects (also known as multilevel or hierarchical) logistic regression models. The models included patient risk factors and random intercepts for each hospital, and allowed analysis of hospitals with a low case load [10,11,19,20]. We built several two-level models. The first model included only the random intercept to estimate inter-hospital variability in overall mortality rate. The second model contained relevant patient comorbidities (identified in a preliminary stepwise logistic regression model), age, gender and STEMI/NSTEMI categorisation. The significance level of entry and removal was set at 0.05. Age and gender were forced into the model. In the final model, we added the presence of a cardiac catheterisation laboratory in the hospital. In addition, we tested the interactions between outcome, AMI phenotype and gender, and then carried out a multilevel analysis on patients with STEMI and NSTEMI separately.

We estimated the odds ratios (ORs) with 95% confidence intervals (CIs) for patient and hospital-related characteristics and the random variance (*σ*^*2*^), which is a measure of inter-hospital variation. We used the area under the ROC curve to assess the discriminative ability of the model in predicting 30-day in-hospital mortality. This area (alternatively named *c*-index) varies from 0.5 to 1, with larger values denoting better model performance. We also reported other goodness of fit indexes, including pseudo *R*^*2*^, the Wald *χ*^*2*^ test, Akaike information criterion (AIC) and Bayesian information criterion (BIC). Then, we calculated the adjusted hospital-specific mortality rates with 95% CIs to identify hospitals with mortality rates significantly different from the adjusted overall mortality rate. Hospital-specific adjusted mortality rates were calculated as the antilogit function of the random intercepts derived from the multilevel logistic regression model which included patient characteristics. The adjusted overall mortality rate was obtained by taking the antilogit function of the intercept estimated from the same model. We expressed these results in terms of RSMRs (risk-standardised mortality rates) [12]. RSMRs with 95% CIs were calculated as hospital-specific adjusted mortality rates over the adjusted overall mortality rate, multiplied by the unadjusted overall mortality rate.

Finally, we used funnel plots as an alternative method for inter-hospital comparison. Funnel plots are scatter plots of unadjusted 30-day in-hospital mortality rates against the number of patients discharged from each hospital [21]. We superimposed on the plot 95% (≈2 standard deviations) and 99.8% (≈3 standard deviations) control limits around the crude overall 30-day in-hospital mortality rate. We chose to use the crude overall rate as the target value in order to make the results from funnel plots comparable to the RSMRs from multilevel analyses.

Statistical analyses were performed using Stata software, version 12 (StataCorp LP, College Station, TX, USA).

## Results

We extracted a total of 6,267 HDRs of patients with AMI from 37 hospitals, 11 of which (29.73%) had a cardiac catheterisation laboratory. Ten of these operated 24-h 7 days a week, one was a 12-h laboratory operating 12 h for 7 days a week, and had an associated 24-h laboratory about 26 km away; 316 HDRs (5.03%) were excluded because their diagnostic code was 410.9.

The study population included 5,832 patients (61.90% male, mean age 72.38 years) admitted to 37 hospitals (Table 1); 114 patients had multiple AMI. The overall proportion of patients with STEMI was 47.15%.

**Table 1:** Patient characteristics according to the presence of a cardiac catheterisation laboratory

**Characteristics**	**All (n=5,832)**	**Presence of cardiac catheterisation lab.**		
		**No (n=2,315)**	**Yes (n=3,517)**	**p-value**
**Age in years, mean [SD]**	72.38 [13.05]	74.62 [12.39]	70.90 [13.26]	<0.001
**Gender (%)**				<0.001
Male	3,610 (61.90)	1,335 (57.67)	2,275 (64.69)	
Female	2,222 (38.10)	980 (42.33)	1,242 (35.31)	
**Hospital stay in days, mean [SD]**	6.79 [5.62]	6.43 [5.54]	7.03 [5.67]	<0.001
**Comorbidities (%)**				<0.001
No	3,918 (67.18)	1,412 (60.99)	2,506 (71.25)	
Yes	1,914 (32.82)	903 (39.01)	1,011 (28.75)	
**Specific comorbidities (%)**				
Hematologic diseases	181 (3.10)	81 (3.50)	100 (2.84)	0.158
History of heart failure	352 (6.04)	184 (7.95)	168 (4.78)	<0.001
Cerebrovascular diseases	273 (4.68)	139 (6.00)	134 (3.81)	<0.001
History of cerebrovascular diseases	261 (4.48)	123 (5.31)	138 (3.92)	0.012
Vascular diseases	259 (4.44)	107 (4.62)	152 (4.32)	0.588
History of vascular diseases	177 (3.03)	77 (3.33)	100 (2.84)	0.293
Chronic nephropathies	470 (8.06)	234 (10.11)	236 (6.71)	<0.001
History of chronic nephropathies	237 (4.06)	128 (5.53)	109 (3.10)	<0.001
History of tumours	167 (2.86)	81 (3.50)	86 (2.45)	0.018
History of diabetes	373 (6.40)	185 (7.99)	188 (5.35)	<0.001
History of hypertensive disease	582 (9.98)	278 (12.01)	304 (8.64)	<0.001
History of other forms of ischemic heart disease	597 (10.24)	294 (12.70)	303 (8.62)	<0.001
History of conduction disorders and cardiac dysrhythmias	288 (4.94)	152 (6.57)	136 (3.87)	<0.001
History of COPD	182 (3.10)	105 (4.54)	77 (2.19)	<0.001
**ST-segment elevation (%)**				<0.001
NSTEMI	3,082 (52.85)	1,509 (65.18)	1,573 (44.73)	
STEMI	2,750 (47.15)	806 (34.82)	1,944 (55.27)	

*COPD* Chronic obstructive pulmonary disease, *SD* standard deviation, *STEMI* ST elevation myocardial infarction, *NSTEMI* Non-ST elevation myocardial infarction.

Patients admitted to hospitals with a cardiac catheterisation laboratory were significantly younger, less comorbid, more frequently male, had STEMI, and had a longer hospital stay (Table 1).

During the study period, 7.99% of patients (interquartile range, 6.45–11.59%) died within 30 days of admission. The 30-day in-hospital mortality rate was significantly higher among patients with STEMI compared with those with NSTEMI (STEMI: 9.89%, interquartile range 5.85–18.18%; NSTEMI: 6.29%, interquartile range 3.73–7.92%; Pearson’s *χ*^*2*^ (d.f.) = 27.46 (1), p < 0.001).

### Multilevel logistic regression analysis

In the overall sample, the multilevel model that included only the hospital-specific random effect showed a significant inter-hospital variation in 30-day in-hospital mortality (*σ*^*2*^ = 0.12, p < 0.001) (Table 2). When patient characteristics, including age, gender, comorbidities and the STEMI/NSTEMI categorisation were added to the model, the hospital variance decreased (*σ*^*2*^ = 0.05, p = 0.084). To account for residual hospital variability, we included in the model the presence of a cardiac catheterisation laboratory. This variable was significantly associated with 30-day in-hospital mortality: specifically, patients from hospitals with a cardiac catheterisation laboratory were 29% less likely to die. This final model had a *c*-index of 0.78, denoting a satisfactory performance in predicting the outcome.

**Table 2:** Odds ratios and 95% CIs for multilevel logistic models estimating 30-day in-hospital mortality in the overall sample

	**Hierarchical null model**	**Hierarchical model without presence of a cardiac catheterisation lab.**	**Hierarchical model with presence of a cardiac catheterisation lab.**
**Patients Characteristics**			
Gender (male *vs*. female)	–	1.10 (0.90–1.36)	1.09 (0.89–1.35)
Age (years)	–	1.09 (1.08–1.10)	1.09 (1.08–1.10)
History of COPD	–	1.99 (1.31–3.01)	1.91 (1.26–2.88)
History of heart failure	–	1.47 (1.07–2.02)	1.46 (1.06–2.00)
History of cerebrovascular diseases	–	1.49 (1.04–2.14)	1.49 (1.04–2.14)
Cerebrovascular diseases	–	1.45 (1.01–2.09)	1.42 (0.99–2.04)
History of tumours	–	2.65 (1.73–4.05)	2.55 (1.67–3.90)
ST-segment elevation (STEMI *vs*. NSTEMI)	–	2.26 (1.83–2.78)	2.31 (1.88–2.84)
**Hospital Characteristic**			
Presence of cardiac catheterisation lab.	–	–	0.71 (0.58–0.87)
**Hospital Variance**			
*σ*^*2*^ (p-value)*	0.12 (<0.001)	0.05 (0.084)	<0.01 (1.000)
**Goodness of fit**			
Pseudo *R*^*2*^	–	0.31	0.32
Wald *χ*^*2*^ (p-value)	–	335.25 (<0.001)	350.58 (<0.001)
AIC	3,261.08	2,843.00	2,836.27
BIC	3,264.30	2,859.11	2,853.99

* p-value from LR (likelihood ratio) test *vs*. logistic regression of *σ*^*2*^ = 1.

*CI* confidence interval, *AIC* Akaike Information Criterion, *BIC* Bayesian Information Criterion.

We found a significant interaction between outcome and AMI phenotype, and a non-significant interaction between outcome and gender. The multilevel analysis was hence carried out separately in the two strata of patients with STEMI and NSTEMI. In patients with STEMI, some residual inter-hospital variation was found, even after adjusting for patient characteristics (Table 3). Incorporating the presence of a cardiac catheterisation laboratory to the model accounted for a significant proportion of hospital variability (the hospital variance decreased to 0.08, p = 0.046). Patients with STEMI from hospitals with a cardiac catheterisation laboratory were 41% less likely to die within 30 days. In contrast, 30-day in-hospital mortality rates of patients with NSTEMI showed no variation among hospitals and the presence of a cardiac catheterisation laboratory did not influence the outcome. The *c*-index for the STEMI and NSTEMI models was 0.76 and 0.79, respectively. According to AIC and BIC, among patients with STEMI the model with patient and hospital characteristics gave the best fit, while among patients with NSTEMI the best model was the one which included only patient characteristics. The analyses of residuals did not reveal the presence of influential observations.

**Table 3:** Odds ratios and 95% CIs for multilevel logistic models estimating 30-day in-hospital mortality by ST-segment elevation

	**STEMI**			**NSTEMI**		
	**Hierarchical null model**	**Hierarchical model without presence of a cardiac catheterisation lab.**	**Hierarchical model with presence of a cardiac catheterisation lab.**	**Hierarchical null model**	**Hierarchical model without presence of a cardiac catheterisation lab.**	**Hierarchical model with presence of a cardiac catheterisation lab.**
**Patients Characteristics**						
Gender (male *vs*. female)	–	1.15 (0.86–1.52)	1.14 (0.86–1.51)	–	1.02 (0.74–1.40)	1.01 (0.74–1.39)
Age (years)	–	1.08 (1.06–1.09)	1.08 (1.06–1.09)	–	1.11 (1.09–1.13)	1.11 (1.09–1.14)
History of COPD	–	2.89 (1.51–5.53)	2.67 (1.40–5.11)	–	1.75 (1.02–3.03)	1.73 (1.00–2.99)
History of diabetes	–	–	–	–	1.65 (1.02–2.67)	1.64 (1.01–2.65)
History of cerebrovascular diseases	–	–	–	–	1.79 (1.08–2.97)	1.80 (1.09–2.98)
Cerebrovascular diseases	–	1.86 (1.13–3.07)	1.81 (1.10–2.98)	–	–	–
History of tumours	–	3.08 (1.70–5.58)	2.93 (1.62–5.32)	–	2.49 (1.34–4.65)	2.50 (1.34–4.67)
Vascular diseases	–	2.05 (1.17–3.59)	2.05 (1.17–3.58)	–	–	–
History of other forms of ischemic heart diseases	–	–	–	–	0.54 (0.33–0.86)	0.54 (0.34–0.86)
History of heart failure	–	–	–	–	1.87 (1.21–2.89)	1.87 (1.21–2.88)
**Hospital Characteristic**						
Presence of cardiac catheterisation lab.	–	–	0.59 (0.42–0.85)	–	–	0.89 (0.65–1.20)
**Hospital Variance**						
*σ*^*2*^ (p-value)*	0.42 (<0.001)	0.17 (0.002)	0.08 (0.046)	<0.01 (1.000)	<0.01 (1.000)	<0.01 (1.000)
**Goodness of fit**						
Pseudo *R*^*2*^	–	0.27	0.29	–	0.37	0.37
Wald *χ*^*2*^ (p-value)	–	162.44 (<0.001)	174.44 (<0.001)	–	151.59 (<0.001)	152.17 (<0.001)
AIC	1,749.24	1,554.80	1,549.84	1,464.31	1,273.26	1,274.64
BIC	1,753.46	1,567.69	1,564.34	1,467.36	1,288.52	1,291.43

* p-value from LR (likelihood ratio) test *vs*. logistic regression of *σ*^*2*^ = 1.

### Comparison of hospital-specific 30-day in-hospital mortality rates with the overall rate

**Figure 1:**
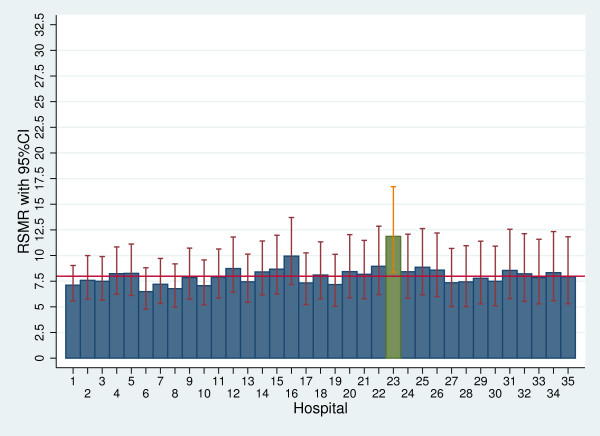
**RSMRs with 95% CIs by hospital.** Red line: overall 30-day in-hospital mortality rate; green bars: significantly higher RSMRs compared with the overall 30-day in-hospital mortality rate. RSMRs with 95% CIs are not reported for hospitals with only one patient (# 36 and 37). Hospitals with a cardiac catheterisation laboratory are 1, 2, 3, 5, 6, 7, 8, 9, 13, 14 and 19. RSMR, Risk-standardised mortality rate.

Figure 1 shows the RSMRs for the overall sample compared with the overall 30-day in-hospital mortality rate. One hospital (#23) displayed a significantly higher mortality rate compared with the overall rate, with a wide confidence interval related to its small number of AMI patients.

**Figure 2:**
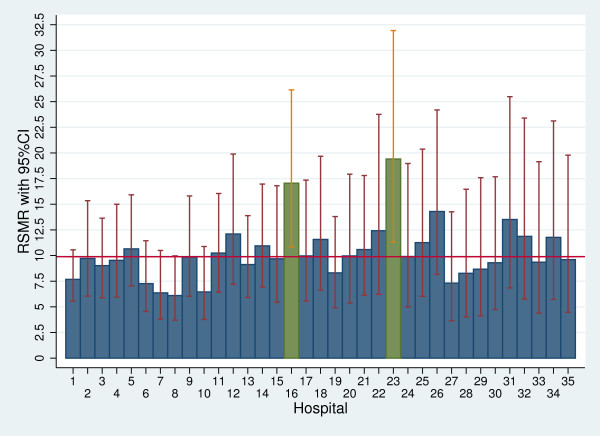
**RSMRs with 95% CIs by hospital (only patients with STEMI).** Red line: overall 30-day in-hospital mortality rate; green bars: significantly higher RSMRs compared with the overall 30-day in-hospital mortality rate. RSMRs with 95% CIs are not reported for hospitals with only one patient (# 36 and 37). Hospitals with a cardiac catheterisation laboratory are 1, 2, 3, 5, 6, 7, 8, 9, 13, 14 and 19.

Stratification by ST-segment elevation revealed two hospitals (#16 and 23) which had mortality rates differing significantly from the overall rate in patients with STEMI (Figure 2), and none in patients with NSTEMI.

**Figure 3:**
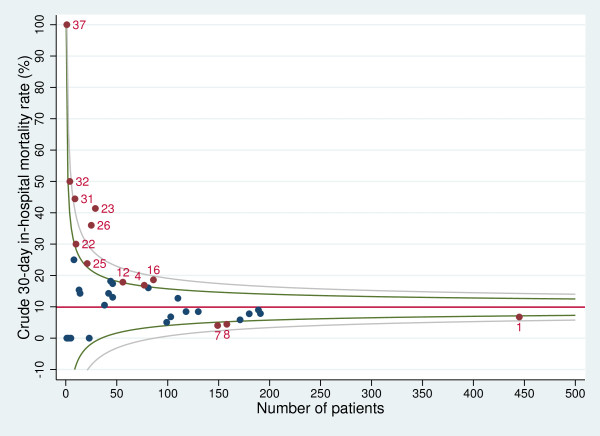
**Funnel plot for unadjusted 30-day in-hospital mortality rate with 95% and 99.8% control limits (STEMI).** Red line: overall 30-day in-hospital mortality rate; green lines: 95% control limits; grey lines: 99.8% control limits; labelled data points: hospitals falling above the upper 95% limit or below the lower 95% limit.

**Figure 4:**
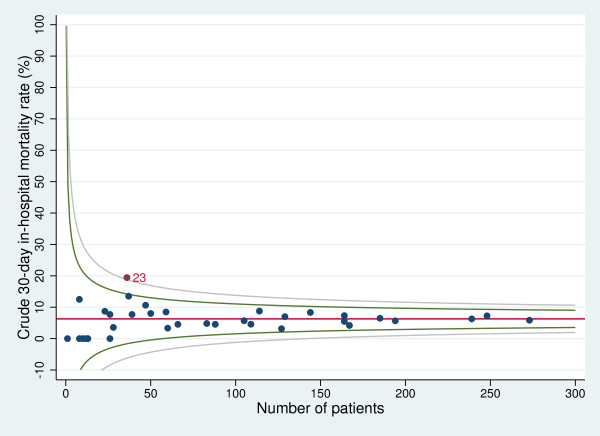
**Funnel plot for unadjusted 30-day in-hospital mortality rate with 95% and 99.8% control limits (NSTEMI).** Red line: overall 30-day in-hospital mortality rate; green lines: 95% control limits; grey lines: 99.8% control limits; labelled data points: hospitals falling above the upper 95% limit or below the lower 95% limit.

Figures 3 and 4 show the funnel plots for STEMI and NSTEMI that depict the relationship between unadjusted mortality rates and the number of discharges. Regarding STEMI, nine hospitals exceeded the upper boundary of the 95% control limit for the crude 30-day in-hospital overall mortality rate. One hospital exceeded the boundaries for both STEMI and NSTEMI.

## Discussion

We compared 30-day in-hospital mortality rates after AMI among 37 hospitals in Tuscany region using administrative data sources. The standardised period of 30 days was chosen to ensure a fair inter-hospital comparison and to prevent differences in length of stay from affecting the measurement. Our results indicate an overall mortality rate of 7.99%, with a significant inter-hospital variability. This variability was largely accounted for by patient characteristics, including the presence or absence of ST-segment elevation, and partly by the presence of a cardiac catheterisation laboratory in the hospital. Stratified analyses on patients with STEMI and NSTEMI revealed that the presence of a cardiac catheterisation laboratory influenced 30-day in-hospital mortality only among patients with STEMI. The power of the predictive models for STEMI and NSTEMI was satisfactory (*c*-index 0.76 and 0.79).

Our 30-day in-hospital mortality rates were within the range of those reported for the US and European population (overall 7.8–16.1, STEMI 5.9–15.0, NSTEMI 3.0–14.0) [2,18,22-26].

The overall proportion of patients with STEMI was 47.15%, which is consistent with the rates reported by Yeh et al. [22], Rogers et al. [27], Ruff et al. [4], and with the recent trend of a decrease in STEMI compared with NSTEMI in Western countries. The large variability among hospitals in the proportion of STEMI and the differential risk related to this condition supports the inclusion of this variable, in addition to comorbidities, in risk adjustment models, to diminish the effect of case mix, in line with the literature [2,28-32]. The classification STEMI/NSTEMI rests upon the validity of coding for these diagnoses. In Tuscany region, the sensitivity and specificity of the STEMI/NSTEMI diagnosis based on the fourth digit of ICD-9-CM AMI codes is in line with US studies based on the National Registry of Myocardial Infarction [33,34].

Regarding the comparison of the outcome of a specific hospital with the regional benchmark, in the multilevel analyses we identified one hospital with mortality significantly higher than the regional rate. Restricting the analyses to patients with STEMI revealed two hospitals with an increased mortality compared with the overall Tuscany rate. Using funnel plots, we identified ten hospitals with a performance significantly poorer than the regional benchmark for STEMI patients. This suggests that funnel plots overemphasise regional discrepancies in the performance of small-volume hospitals. We argue that inter-hospital comparisons should preferably be performed using multilevel analyses, because they “minimize the impact of small sample sizes by borrowing power across the entire sample and ensuring that hospitals with small numbers are not identified as outliers because of adverse events in just a few patients” [10]. Moreover, our data show the importance of adjusting for patient characteristics.

Regarding the impact of organisational characteristics on outcome, we found that the presence of a cardiac catheterisation laboratory in the hospital accounted for a lower 30-day in-hospital mortality only in patients with STEMI. Our results are consistent with two New Zealand studies, showing that patients hospitalised for ACS, if admitted to a hospital without a cardiac catheterisation laboratory, suffered delays in diagnosis and subsequent revascularisation [35,36]. In contrast, evidence from other studies suggests that the risk of death in patients with STEMI, admitted to a hospital equipped with a cardiac catheterisation laboratory, does not differ significantly from that of patients admitted to a hospital without the laboratory [37,38]. A possible explanation of this lack of difference is that a reduction in the risk of death is achieved when reperfusion is performed within a short time, either in the same hospital or by transferring the patient to another facility. This is corroborated by a meta-analysis carried out on patients with STEMI [39]. In addition, an Italian study found that implementing a "reperfusion network" at the regional level effectively reduced the rates of hospital mortality and major cardiovascular and cerebrovascular events in the year following the index admission [40]. A recent study showed that hospitals with high-quality medical and interventional management had better outcomes [41], and another report differentiated high-performing hospitals by an organisational culture that supported efforts to improve AMI care [42]. Unfortunately, information on medical treatments and door-to-balloon time is not available in the hospital discharge database and we cannot provide data to support or disprove these findings. In our study, although some of the hospitals without a cardiac catheterisation laboratory had good outcomes, the hospitals that showed the best outcomes proved to be those equipped with a cardiac catheterisation laboratory.

### Limitations

Our results should be interpreted bearing in mind the strengths and weaknesses of the use of routine administrative databases for inter-hospital comparison of 30-day in-hospital mortality after AMI. While hospital discharge records are widely available at reasonable cost, they do not provide clinical data and information on medical treatments that can influence inter-hospital differences [43]. It is therefore possible that differences in the severity of cases in this study are not accounted for. However, evidence from a large Canadian study [8] suggested that the use of detailed clinical data for inter-hospital comparison of AMI mortality rates does not result in a reduction in the number of hospitals with adjusted AMI mortality rates higher than expected.

It is also possible that comorbidity is underreported in some hospitals, which may lead to an overestimation of adjusted death rates for those hospitals. Another potential source of bias may be that some hospital recorded secondary diagnoses more accurately than others, which may have an impact if hospitals that code comorbidities poorly also provide better or worse care than other hospitals. Last, we chose to use multilevel logistic regression analyses with a dichotomous outcome. Other models including time to death, such as frailty survival models, would have been possible. However, given the lack of information on deaths occurring at home, we preferred to use a simpler model.

## Conclusions

We have shown that it is possible to use routinely collected administrative data to predict, with satisfactory power, the short-term risk of death in AMI patients, and to highlight inter-hospital differences. The distinction between STEMI and NSTEMI proved to be useful to identify inter-hospital variations, which affected only the STEMI subgroup.

Our results have important implications in terms of health care policy. We confirm that the use of administrative data sources for monitoring mortality routinely is potentially useful in quality assessment and improvement efforts. In particular, the variability in adjusted mortality rates among hospitals suggests the need to develop efficient auditing activities to detect critical aspects of the organisation. Our data showed the beneficial effect of the presence of a cardiac catheterisation laboratory in the hospital, and support the view that timely reperfusion may significantly decrease short-term mortality in patients with AMI. We suggest that an efficient reperfusion network can achieve this goal.

## Competing interests

The authors declare that they have no competing interests.

## Authors’ contributions

CS and MPF contributed to the conception of the study, and acquisition and interpretation of data; SM, CS and JL carried out the statistical analyses. All authors read and approved the final manuscript.

## Pre-publication history

The pre-publication history for this paper can be accessed here:

http://www.biomedcentral.com/1471-2288/12/170/prepub
